# Socioeconomic Factors and Prevalence of Functional Dependence Assessed by the Barthel Index and the Lawton-Brody Scale in the Older Population Residing in Block Hazratbal, Srinagar, India

**DOI:** 10.7759/cureus.112501

**Published:** 2026-07-12

**Authors:** Ayman Nisar, Talha Malik, Tazean Z Malik, S. M. Salim Khan

**Affiliations:** 1 Community Medicine, Government Medical College, Srinagar, IND; 2 Medicine, Government Medical College, Srinagar, IND; 3 Community Medicine, Sher-i-Kashmir Institute of Medical Sciences, Srinagar, IND

**Keywords:** activities of daily living, functional dependence, instrumental activities of daily living, older people, vulnerable population

## Abstract

Background

Most developing countries face a growing number of older populations because of a decrease in fertility and an increase in longevity. The older age stage (≥ 60 years) is a period in which people are prone to chronic diseases and physical and mental disabilities, which probably restrict their functional independence. Functional status refers to a person's ability to perform tasks that are required for living.

Objective

The objective of this study was to determine the prevalence of functional dependency and its associated factors among the older population in Block Hazratbal, Srinagar, India.

Methodology

A cross-sectional study was conducted in Block Hazratbal, Srinagar, from August 2024 to March 2025. The study participants included older individuals (age ≥60 years) who had been residing in the Block for at least the past six months. The presence of functional dependence was assessed using the Barthel Index for activities of daily living (ADL) and the Lawton-Brody Scale for instrumental activities of daily living (IADL).

Results

Out of a total of 257 participants, females comprised 58.4% of the population, and the rest were males. The mean age of the participants was 66.33±5.2 years. The prevalence of any functional dependence as per the Barthel index was 40.8%, and moderate to total dependency was seen in 35.4% of participants. More females than males were functionally dependent while performing different IADL tasks, and females also expressed financial dependence on their families.

Conclusion

One in three older persons in the study experienced a moderate to severe level of physical dependency. This raises the question of whether home care and geriatric services in India at the primary, secondary, and tertiary levels are adequately scaled to provide quality care for these individuals.

## Introduction

The global population is undergoing a rapid demographic transition, with a notable rise in the proportion of older individuals. According to the World Health Organization (WHO), the number of people aged 60 years and above is expected to increase from 12% in 2015 to 22% by 2050, doubling in absolute terms from 900 million to 2 billion people worldwide. Many developing countries are facing a public health challenge in the form of an aging population due to decreasing fertility rates and increasing longevity [1]. The percentage of older people in India has been increasing rapidly in recent years, and this trend is likely to continue. The proportion of the population aged 60 and over is projected to rise from 10.5% in 2022 to 20.8% by 2050. By the end of the century, the older population will account for over 36% of the nation's total population [2]. According to the Ministry of Statistics and Implementation, the older population in India is projected to rise to 319 million by 2050 from 138 million in 2021 [3].

Older age (≥ 60 years) is a time when individuals are susceptible to chronic diseases, and physical and mental disabilities often limit their functional independence [4]. The capacity to perform activities of daily living (ADL) is a crucial factor in determining the level of dependency experienced by older individuals. Functional dependence in older adults is a critical indicator of health and quality of life. It refers to the need for assistance in performing essential tasks of daily living, including both basic activities of daily living, such as bathing, dressing, and eating, and instrumental activities of daily living (IADLs), like using transportation, managing finances, and preparing meals [5,6]. Competence in skills such as shopping, cooking, and managing finances is required for independent living. Because IADL function is usually lost before ADL function (such as bathing, eating, and using the toilet), assessment of IADLs may identify incipient decline - physical, cognitive, or both - in an older adult who might otherwise appear capable and healthy [7]. Loss of functional independence not only increases the burden on caregivers and the healthcare system but also adversely affects the psychological and social well-being of older individuals. Assessing functional dependence is essential for identifying vulnerable older individuals, planning long-term care, and designing public health interventions aimed at improving their quality of life.

In India, nearly one-fifth of the older population has at least one ADL limitation [2]. According to the Longitudinal Aging Study in India (LASI), 2017-2018 report, in Jammu and Kashmir, the proportion of older individuals experiencing restrictions in activities of daily living stands at 21.2% [8]. Several studies in India have highlighted varying levels of functional dependence among older persons, influenced by factors such as age, gender, socio-economic status, chronic illnesses, and environmental conditions [9-12]. However, there is a paucity of data from geographically distinct areas like Jammu and Kashmir, which is characterized by unique socio-cultural dynamics and challenges in accessing health services that may influence the aging experience and the degree of functional independence.

Given these factors, it is imperative to understand the functional health status of the older population in this region to inform public health planning and resource allocation. This study, therefore, aims to assess the level of functional dependence among the older population residing in Block Hazratbal, Srinagar, and identify socio-demographic and health-related correlates.

## Materials and methods

Study design and duration

A community-based cross-sectional study was conducted over a period of eight months, from August 2024 to March 2025.

Study setting

The study was conducted in Block Hazratbal, a semi-urban administrative unit in Srinagar district, Jammu and Kashmir, India. Block Hazratbal has an estimated population of approximately 80,000 individuals. The block is primarily served by the subdistrict Hospital (SDH), which provides 24/7 outpatient and emergency services. The hospital conducts a dedicated weekly geriatric outpatient clinic, which addresses the specific health needs of the older population in the region. In addition, as part of their postgraduate training, the residents conduct weekly domiciliary visits to the geriatric population in the area.

Study population

The target population comprised older individuals aged 60 years and above who had been permanent residents of Block Hazratbal for at least six months prior to the survey.

Exclusion Criteria

Older persons with any previously diagnosed malignancy, those who were bedridden or unable to stand unsupported due to extreme physical debility, and individuals with diagnosed severe cognitive impairment preventing meaningful participation were excluded from the study. The exclusion criteria were specifically aligned with the study's primary outcomes. Bedridden individuals and those unable to stand unsupported were excluded to prevent a statistical 'floor effect', as their profound physical limitations would inevitably yield near-zero scores, leaving no room to measure functional changes. Individuals with severe cognitive impairment were excluded because cognitive deficits lead to functional dependence via neurological rather than physical mechanisms, which would confound the assessment of physical functional trajectories. Finally, patients with a prior malignancy were excluded because cancer-related complications (e.g., cachexia, treatment fatigue) introduce acute, fluctuating changes in ADL and IADL scores that do not reflect standard geriatric functional decline. 

Sample size

The sample size was calculated using Cochran's formula for prevalence studies:

$N=\frac{Z^{2}p(1-p)}{d^{2}}$

Where N is the total sample size, Z=1.96 at 95% confidence interval, p=21.2%, and d=5% absolute error.

A sample size of 257 was obtained based on the proportion of functional dependency of 21.2% in Jammu and Kashmir, as reported in the Longitudinal Aging Study in India (LASI) [8].

Sampling technique

All eligible older individuals encountered during the weekly household visits in the study area were approached serially until more than the minimum required sample size was achieved (taking into account improperly/partially filled questionnaires). The first household was selected randomly by a computer-generated random number from the list of households collated by the field workers in the Block. Each subsequent household was selected by moving to the immediate next-door household. If the house was found locked, we moved to the next house serially. All eligible older individuals in the household were approached for inclusion in the study. 

Study tools

The data were collected using a pre-tested, semi-structured questionnaire, which consisted of demographic, medical, and functional assessment sections. Functional dependency was assessed using dependence in ADL and IADL.

Activities of Daily Living

Activities of daily living (ADL) was measured using the Barthel Index for ADL. This tool assesses an individual's ability to perform 10 basic self-care tasks, including feeding, bathing, dressing, toileting, mobility, and grooming. The scoring for each activity in the Barthel Index is between 0 and 10, where full independence is given a score of 10 and needing some assistance in performing the activity is scored as 5. A score of zero is given if the person is completely unable to perform the activity. The scores range from 0 (fully dependent) to 100 (fully independent), with higher scores indicating greater independence. The level of functional dependence as per the Barthel Index is categorized as fully independent (score 96-100), slight dependence (91-95), moderately dependent (61-90), severe dependence (21-60), and totally dependent (score 0-20) [13]. For analysis, the Barthel index was dichotomized into two groups: independent (score 91-100) and moderate to totally dependent (score 0 to 90). With this, the study intentionally filters out individuals with subclinical, slight, or transient impairments (e.g., minor assistance needed only for negotiating stairs or occasional bowel/bladder tracking lag). Consequently, the 0 to 90 cohort aggregates patients experiencing moderate, severe, and total dependency, thereby isolating a clinically distinct group that faces a genuine, sustained caregiver burden and a lack of daily physical autonomy.

Instrumental Activities of Daily Living

Instrumental activities of daily living (IADL) was measured by the Lawton-Brody IADL scale. This scale measures an individual's ability to perform eight more complex activities necessary for independent living, such as handling finances, using transportation, managing medications, and preparing meals. Women are scored on all eight domains, but for men, the domains of food preparation, housekeeping, and laundering are excluded. Each activity is rated dichotomously as zero or one based on the level of independence in performing the activity, and the total scores across all domains are added. The total scores range from 0 to 8 (for women) and 0 to 5 (for men); lower scores indicate increased dependence [6,14]. The final scores are presented as a percentage of the optimal function. For example, a score of 4 out of 5 would represent 80% function (80% independence and 20% dependence). For the present study, questions on all eight domains were asked of both men and women to compare apparent dependency across domains; however, scores were calculated using the traditional gender-based scoring method, as most Indian households still subscribe to traditional gender roles. For analysis, the Lawton-Brody index was dichotomized as independent (100% functionality) and dependent (<100% functionality)[15].

Both scales are available in the public domain for non-commercial, educational and research purposes. Both tools were administered in the local languages (Kashmiri/Urdu) after translation and back-translation to ensure consistency and validity. The socio-demographic data, consisting of age, gender, occupation, income, etc., were also collected using a self-designed questionnaire. Socio-economic status was calculated using the Modified B.G Prasad scale 2025 [16]. For co-morbid conditions, only those individuals who had already been diagnosed with a particular condition (hypertension, diabetes mellitus, hypothyroidism, osteoporosis, arthritis, etc.) were included.

Data collection

Individual face-to-face assessments were conducted by two trained investigators, strictly in accordance with the indices' standards, at the participants' residences. Pilot testing of the study tools was done to ensure familiarisation with the study procedures. The investigators worked as a team, and for each participant, one investigator assessed the Barthel Index while the other assessed the Lawton-Brody Index for all participants. The purpose of the study was explained to each participant in their native language, and written informed consent was obtained. Privacy and confidentiality were strictly maintained throughout the data collection process.

Data analysis

All collected data were entered in Microsoft Excel (Microsoft, Redmond, Washington) by the interviewers (authors AN and TM). Data were cleaned and then analyzed using SPSS Statistics version 25 (IBM Inc., Armonk, New York) (authors TZM and SMSK). A descriptive analysis was performed using mean/standard deviation for continuous variables and counts/proportions for categorical variables. Differences between categorical variables were analyzed using the chi-squared test/Fisher's exact test. Wherever applicable for chi-square tests, Bonferroni correction was used for adjustment. The association between two categorical variables was analyzed using the odds ratio (OR), which compares the odds of the occurrence of an event in one group versus another group. Binary logistic regression analysis was conducted to identify factors associated with functional dependence. A p-value < 0.05 was considered statistically significant.

Ethical considerations

Ethical clearance for the study was obtained from the Institutional Review Board before the commencement of the study. Written informed consent was obtained from all participants before enrollment. Participation was entirely voluntary, and respondents were free to withdraw at any stage without any consequences. Anonymity and confidentiality of personal data were assured throughout the research process. The study procedures were explained to the participants in detail in their local language (Kashmiri/Urdu). Participants were also provided with a patient information sheet in the local language detailing the study procedures. Participants and their caregivers were encouraged to clarify their doubts, if any, and then sign the informed consent form. For illiterate participants, the caregiver's assent was recorded in addition to the participant's thumb impression.

## Results

A total of 265 participants were approached, of whom 257 were enrolled in the study (non-response rate = 3.11%). The mean (±SD) age of the study participants was 66.33 ± 5.2 years. Out of the total participants, 107 (41.6%) were males, and the rest were females. The majority of the participants were in their sixties (n=186, 72.4% of the total) and presented a high comorbidity (n=196, 76.3%). Although many were unemployed (n=136, 52.9%) and had a low educational level (n=136, 52.9% were illiterate), these individuals had a medium socioeconomic status (n=156, 60.7% belonging to the middle class). The other sociodemographic details have been described in Table 1.

**Table 1:** Socio-demographic variables ^a^Kuppuswamy's educational classification; ^b^Modified BG Prasad scale (2025)

Sociodemographic variable	Frequency (percentage)
Age group (years)	
60-69	186 (72.4%)
70-79	67 (26.1%)
80 and above	4 (1.6%)
Gender	
Male	107 (41.6%)
Female	150 (58.4%)
Education^a^	
Illiterate	136 (52.9%)
Primary	36 (14.0%)
Middle	44 (17.1%)
High school	28 (10.9%)
Higher secondary	4 (1.6%)
Graduate	9 (3.5%)
Socioeconomic status^b^	
Upper	0
Upper-middle	83 (32.3%)
Middle	156 (60.7%)
Lower-middle	18 (7.0%)
Lower	0
Occupation	
Unemployed	136 (52.9%)
Retired from service and now continuing occupation	37 (14.4%)
Self employed	46 (17.9%)
Agricultural	38 (14.8%)
Co-morbid conditions	
Yes	196 (76.3%)
No	61 (23.7%)

**Figure 1:**
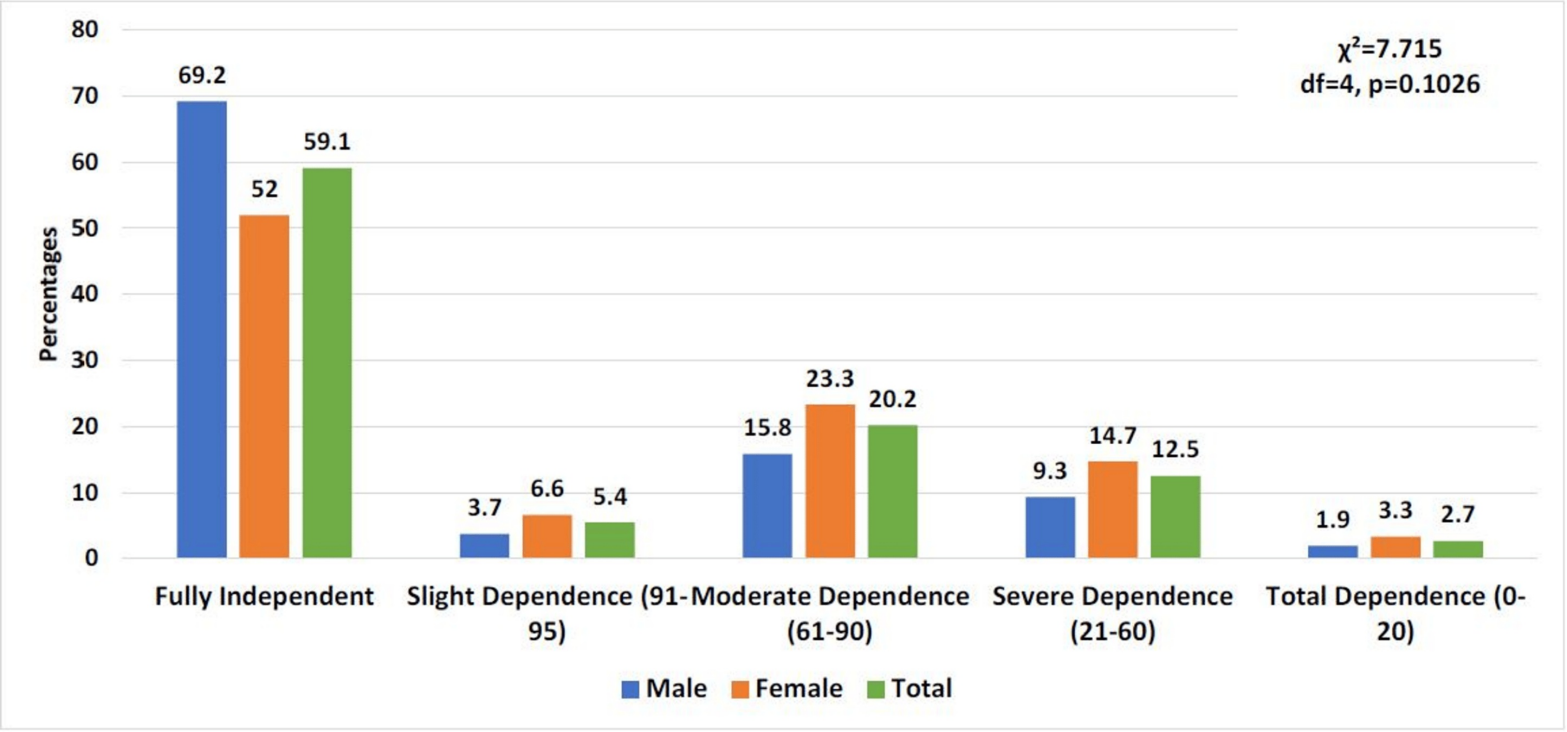
Level of dependency as per the Barthel Index (percentages)

In the present study, the number (%) of totally independent individuals was 152 (59.1%), while the remaining individuals had varying levels of dependency (n=105, 40.8%). The prevalence of functional dependence was 40.8% according to the Barthel Index. Table 2 depicts the different levels of functional dependency categorized according to the gender of the participant. To account for multiple testing across five Barthel Index categories, a Bonferroni correction was applied, setting the statistical significance threshold at p < 0.01. As shown in Figure 1, more females than males experienced dependency in each category of the Index; however, it was not statistically significant (χ²=7.715, df=4, p=0.1026). 

**Table 2:** Prevalence and categorization of functional dependence as per the Barthel Index p-value <0.01 is significant (based on the Bonferroni correction of the chi-squared statistic)

Barthel Index categories	Total (n=257)	Male (n=107)	Female (n=150)	chi-squared, p-value
Full independence (96-100)	152 (59.1%)	74 (69.2%)	78 (52.0%)	7.579, p=0.0059
Slight dependence (91-95)	14 (5.4%)	4 (3.7%)	10 (6.6%)	1.035, p=0.3088
Moderate dependence (61-90)	52 (20.2%)	17 (15.8%)	35 (23.3%)	2.136, p=0.1438
Severe dependence (21-60)	32 (12.5%)	10 (9.3%)	22 (14.7%)	1.615, p=0.2037
Total dependence (0-20)	7 (2.7%)	2 (1.9%)	5 (3.3%)	0.503, p=0.4780

The Lawton-Brody IADL scale indicates the level of dependence in instrumental activities of daily living, expressed as a percentage. Out of the total participants, 64 (24.9%) expressed an IADL independence of less than 25%, while 94 (36.6%) were totally independent. The number of males who expressed a functional independence of less than 25% was 12 (11.2%), while the number of such females was 52 (34.7%) (χ²=18.294; p<0.001). Table 3 depicts the level of dependency in IADL in the study population, showing the different chi-square statistics. The overall Pearson chi-square test was highly significant (χ²=58.46, df=4, p<0.0001), indicating that the level of optimal functionality is strongly associated with the participant's gender. Post-hoc analysis using adjusted standardized residuals also revealed a distinct pattern between the genders. 

**Table 3:** Percentage of optimal functionality as per the Lawton-Brody scale *Males were scored on only five domains out of eight p-value <0.01 is significant (based on the Bonferroni correction of the chi-squared statistic)

Percentage of independence in instrumental activities of daily living*	Total (N=257)	Males (n=107)	Females (n=150)	chi-squared, p-value
<25%	64 (24.9%)	12 (11.2%)	52 (34.7%)	18.294, p<0.0001
25-49%	26 (10.1%)	16 (15.0%)	10 (6.7%)	4.697, p=0.030
50-74%	35 (13.6%)	7 (6.5%)	28 (18.7%)	7.773, p=0.0053
75-99%	38 (14.8%)	8 (7.5%)	30 (20.0%)	7.743, p=0.0054
100%	94 (36.6%)	64 (59.8%)	30 (20.0%)	42.506, p=<0.0001

**Figure 2:**
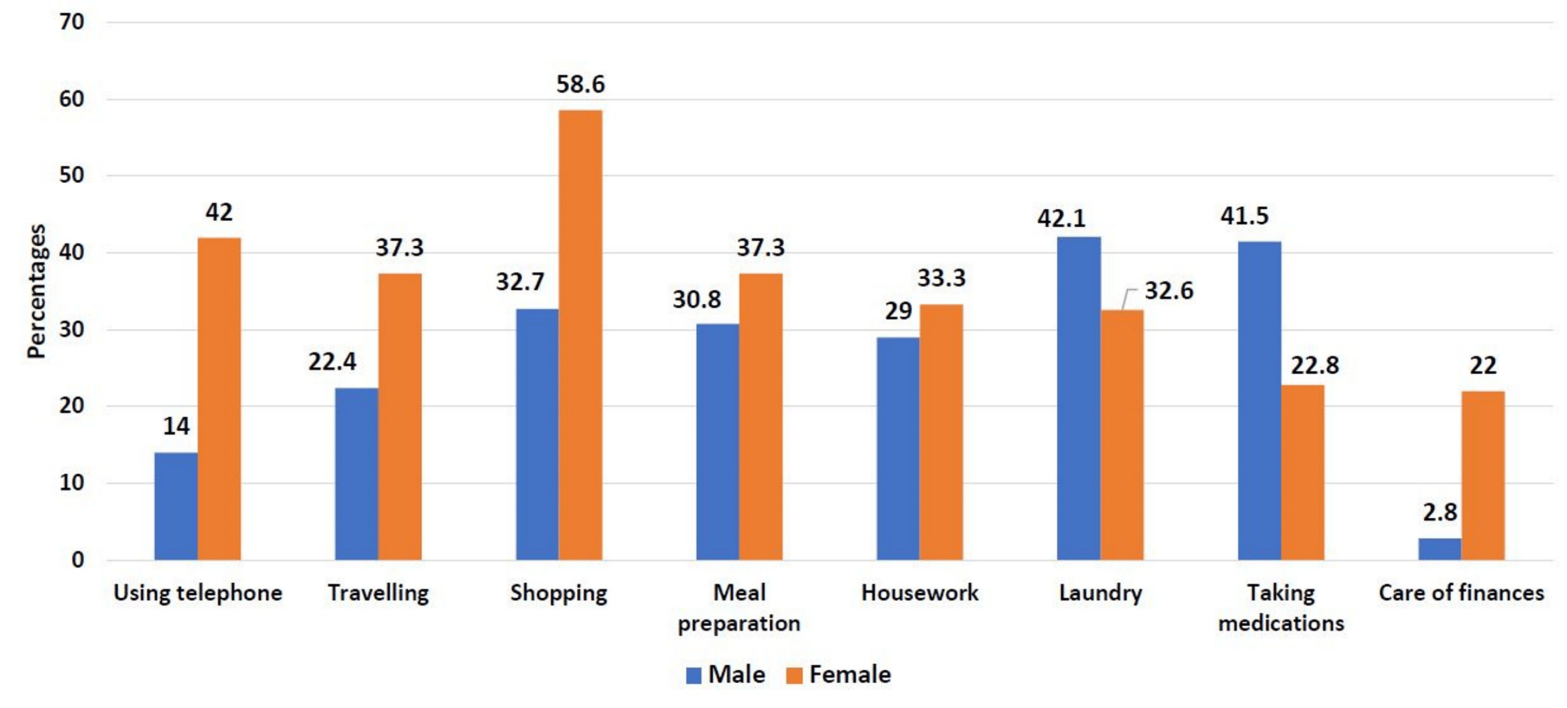
Functional dependency as per the Lawton Brody IADL scale (percentages) IADL - instrumental activities of daily living

Dependency in IADL was relatively higher among females than males across most domains. Regarding medication intake, men are significantly more dependent than women (χ²=7.663, p=0.0056), as shown in Table 4. Also, although the difference is not significant, the percentage of men who depend on others for laundry work is higher than that of women, as shown in Figure 2 and Table 4.

**Table 4:** Participant dependency for individual IADLs using the Lawton-Brody IADL scale *Traditionally considered female domains; ^#^Those who answered in the affirmative when asked whether they were currently taking any medications IADL - instrumental activities of daily living

Activity	Dependents		chi-squared, p-value
	Male (n=107)	Female (n=150)	
Using telephone	15 (14.0%)	63 (42.0%)	23.041, p<0.0001
Traveling places beyond walking distance	24 (22.4%)	56 (37.3%)	6.445, p=0.0111
Shopping	35 (32.7%)	88 (58.6%)	16.795, p<0.0001
Meal preparation*	33 (30.8%)	56 (37.3%)	1.158, p=0.2818
Housework*	31 (29.0%)	50 (33.3%)	0.548, p=0.4590
Laundry*	45 (42.1%)	49 (32.6%)	2.364, p=0.1242
Taking medications (Males=77, Females=118)^#^	32 (41.5%)	27 (22.8%)	7.663, p=0.0056
Care of finances	3 (2.8%)	33 (22.0%)	19.030, p<0.0001

The association between functional dependency (as measured by the Barthel index) and the independent variables was assessed using the chi-squared test for categorical variables. Functional dependency was categorized as a dichotomous variable, with participants scoring 90 or less designated as dependent (moderate, severe, and total dependence categories), and the rest as independent. The moderate to total functional dependency was found to have a significant association with female gender (χ^2^=5.508, p=0.018), age of the participant (χ^2^=148.519, p<0.0001), presence of co-morbid conditions (like diabetes mellitus, hypertension, hypothyroidism, etc.; Appendix) (χ^2^=32.386, p<0.0001), education (χ^2^=34.299, p<0.0001), and occupation of the participant (χ^2^=6.592, p=0.010) (Table 5). The association between age and functional dependency should be interpreted with caution as the Odds' ratio is very high and the 95% CI is very wide. 

**Table 5:** Association of functional dependency (Barthel Index) with various variables ADL - activities of daily living

Variable		Functional dependency (ADL) (N=257)		chi-squared, p-value	OR (95% CI)
		Independent n=166 (64.6%)	Dependent n=91 (35.4%)		
Gender	Male	78 (72.9%)	29 (27.1%)	5.508, p=0.018	1.89 (1.11-3.24)
	Female	88 (58.7%)	62 (41.3%)		
Age (years)	60-69	162 (87.1%)	24 (12.9%)	148.519, p<0.0001 (Fisher’s exact test)	113.06 (37.78-338.34)
	70 and above	4 (5.6%)	67 (94.4%)		
Co-morbid conditions	No	58 (95.1%)	3 (4.9%)	32.386, p<0.0001 (Fisher’s exact test)	15.75 (4.77-52)
	Yes	108 (55.1%)	88 (44.9%)		
Occupation	Employed	88 (72.7%)	33 (27.3%)	6.592, p=0.010	1.98 (1.17-3.35)
	Unemployed	78 (57.4%)	58 (42.6%)		
Education	Illiterate	64 (47.1%)	72 (52.9%)	34.299, p<0.0001	8.1 (3.0-21.89)
	Primary-middle	66 (82.5%)	14 (17.5%)		1.53 (0.51-4.58)
	High school and above	36 (87.8%)	5 (12.2%)		1
Socio-economic status	Upper-middle	56 (67.5%)	27 (32.5%)	2.305, p=0.316	1
	Middle	96 (61.5%)	60 (38.5%)		1.3 (0.74-2.27)
	Lower-middle	14 (77.8%)	4 (22.2%)		0.59 (0.18-1.97)

For the association between functional dependency, measured by the Lawton-Brody index, and patient attributes, the index was categorized as a dichotomous variable, with participants with 100% functionality designated as independent and the rest (0% to 99%) as dependent. Table 6 depicts this association, in which all the independent variables were significantly associated with the dependent variable.

**Table 6:** Association of functional dependency (Lawton-Brody) with various variables IADL - instrumental activities of daily living

Variable		Functional dependency (IADL) (N=257)		chi-squared, p-value	OR (95% CI)
		Dependent n=163 (63.4%)	Independent n=94 (36.6%)		
Gender	Male	43 (40.2%)	64 (59.8%)	40.973, p<0.0001	5.95 (3.41-10.38)
	Female	120 (80.0%)	30 (20.0%)		
Age (years)	60-69	97 (52.1%)	89 (47.8%)	36.742, p<0.0001	12.11 (4.67-31.43)
	70 and above	66 (92.9%)	5 (7.0%)		
Co-morbid conditions	No	23 (37.7%)	38 (62.3%)	21.377, p<0.0001	4.13 (2.26-7.55)
	Yes	140 (71.4%)	56 (28.6%)		
Occupation	Employed	51 (42.1%)	70 (57.9%)	42.899, p<0.0001	6.41 (3.62-11.32)
	Unemployed	112 (82.4%)	24 (17.6%)		
Education	Illiterate	118 (86.8%)	18 (13.2%)	70.706, p<0.0001	17.88 (7.64-41.85)
	Primary-middle	34 (42.5%)	46 (57.5%)		2.02 (0.89-4.58)
	High school and above	11 (26.8%)	30 (73.2%)		1
Socio-economic status	Upper-middle	43 (51.8%)	40 (48.2%)	7.804, p=0.020	1
	Middle	106 (67.9%)	50 (32.1%)		1.97 (1.14-3.41)
	Lower-middle	14 (77.8%)	4 (22.2%)		3.26 (0.99-10.72)

The variables found statistically significant (p<0.05) in the univariate analysis (Table 5) were then fitted for a binary logistic regression model. Multicollinearity was also assessed. Adjusted Odds Ratios (aORs) and 95% Confidence Intervals (CI) were calculated for all such variables (Table 7). The final logistic regression model demonstrated a good fit to the data as indicated by a non-significant Hosmer-Lemeshow test (χ^2^=14.034, d.f=8, p=0.081). The Nagelkerke R^2^ for the model was 0.698. Female gender, illiteracy, presence of co-morbid conditions, and unemployment were found to increase the odds of functional dependency in the population significantly.

**Table 7:** Logistic regression model for correlates of functional dependency (Barthel Index) Nagelkerke R^2^ = 0.698

Variable		β	S.E	Wald χ²	aOR	95% CI	p-value
Gender	Male	Ref.	-	-	1.000	-	-
	Female	1.736	0.739	5.525	5.674	1.330-24.154	p=0.018
Age	60-69	Ref.	-	-	1.000	-	-
	70 and above	4.323	0.5835	54.908	75.414	24.030-236.668	p<0.001
Education	High school and above	Ref.	-	-	1.000	-	-
	Illiterate	1.340	0.507	6.985	3.819	1.413-10.316	p=0.008
	Primary-middle	1.423	0.907	2.461	3.421	0.701-24.55	p=0.116
Occupation	Employed	Ref.	-	-	1.000	-	-
	Unemployed	1.614	0.807	4.000	5.022	1.032-24.427	p=0.045
Co-morbid conditions	No	Ref.	-	-	1.000	-	-
	Yes	1.174	0.409	8.239	3.234	1.451-7.211	p=0.004

However, due to sparse data in one of the age categories (age=70 years and above), to minimize sparse-data bias, penalized regression was also utilized. Firth's penalization method was selected to obtain reliable parameter estimates in the presence of quasi-complete separation (Table 8). The overall model fit was highly statistically significant, as indicated by a penalized likelihood ratio test (χ^2^=167.332, d.f=6, p<0.001). As seen in Table 8, female gender, age 70 years, illiteracy, unemployment, and presence of co-morbid conditions remained statistically significant factors associated with functional dependency.

**Table 8:** Firth's penalized regression for correlates of functional dependency (Barthel Index) Likelihood ratio test=167.332, d.f=6, p<0.001 PLRTχ² - penalized likelihood ratio test chi-squared statistic

Variable		β	S.E	PLRT χ²	aOR	95% CI	p-value
Gender	Male	Ref.	-	-	1.000	-	-
	Female	1.722	0.674	6.527	5.595	1.493-20.968	p=0.010
Age	60-69	Ref.	-	-	1.000	-	-
	70 and above	4.110	0.544	57.010	61.107	22.780-204.741	p<0.001
Education	High school and above	Ref.	-	-	1.000	-	-
	Illiterate	1.236	0.342	9.740	3.444	1.760-6.728	p=0.001
	Primary-middle	1.112	0.814	1.867	3.040	0.616-14.990	p=0.172
Occupation	Employed	Ref.	-	-	1.000	-	-
	Unemployed	1.579	0.753	4.397	4.850	1.108-21.218	p=0.036
Co-morbid conditions	No	Ref.	-	-	1.000	-	-
	Yes	0.979	0.321	9.302	2.331	1.418-4.993	p=0.002

## Discussion

The present study among 257 older residents of Hazratbal showed a mean age of 66.33± 5.2 years, with females constituting 58.4% of the participants. This female predominance reflects the well-recognized "feminization of aging", a demographic trend observed across India due to higher female life expectancy. National demographic reports such as the Census of India (2011) and publications by the United Nations Population Fund have consistently shown a higher proportion of women in older age groups [17,18]. Similar patterns have been documented in community-based geriatric studies from different parts of India, including the work of Lena et al. in coastal South India and Tiwari et al. in northern India, both of which reported a greater proportion of older women in their samples [19,20]. This gender composition is not merely demographic; it has practical implications, as women often experience greater morbidity, economic dependency, and social vulnerability in later life, all of which may influence functional outcomes.

The present study found that 35.4% of participants had moderate to total ADL dependence according to the predefined Barthel Index cut-off. The proportion of older individuals with ADL limitation in this study is comparable to findings from large national datasets. The LASI wave 1 (2017-18) reported that approximately one-fifth of older adults in India experience at least one ADL limitation, with higher prevalence in advanced age groups and among women [8]. State-level estimates from LASI indicate a substantial burden of functional limitation in northern states, including Jammu and Kashmir. Similarly, using the LASI data, Pengpid et al. reported that 28-32% of older adults in India had functional limitations, with significant rural-urban and gender disparities [21]. The slightly higher prevalence observed in the present study may reflect regional sociodemographic characteristics, differences in measurement categorization, and the community-based sampling approach. The relatively higher proportion of independent individuals may reflect the continued presence of family-based support systems and multigenerational households, which are still common in Kashmiri society.

Gender differences in functional dependency were marked in our study, with females demonstrating significantly higher ADL and IADL dependence. This finding is consistent with national and international literature. Analyses from LASI have shown that older women are more likely than men to report both ADL and IADL limitations [8,21]. Globally, studies from the WHO Study on Global Aging and Adult Health (SAGE) also demonstrate higher disability prevalence among women, even after adjusting for age and socioeconomic factors [22]. Several explanations have been proposed: women generally have longer life expectancy, thereby surviving into ages with greater disability risk; they have a higher prevalence of musculoskeletal disorders such as osteoporosis and arthritis; and they often experience lifelong socioeconomic disadvantage, including lower educational attainment and financial dependence [22,23]. Additionally, lower literacy levels and limited financial independence among older women, especially in traditional settings, can directly influence IADL domains such as managing finances, transportation, and medication handling.

The fact that more men than women are dependent when it comes to taking medication could be explained in terms of the maintenance of social roles. Although overall dependence on instrumental activities is more common among females, a distinct pattern emerges in this dependence, such that males are less dependent in activities traditionally associated with a masculine role, such as travelling, communicating with others by telephone, or managing bank accounts, while females tend to take on, to a greater extent than men, activities more associated with family care, such as shopping, meal preparation, and laundry. This division of roles revealed by the results highlights the persistence of ancestral roles in today's society. Thus, it is important to recognize that some IADL components are shaped by culturally defined gender roles; in many households, certain activities may not have been performed by women historically, which could influence scoring and apparent dependency. And perhaps this could explain why, in this study, meal preparation did not show significant differences between males and females.

Advancing age showed a strong association with dependency in univariate analysis, which further persists, showing statistical significance after adjustment in the multivariable model. Age-related decline in functional capacity is biologically plausible and widely documented. Fried et al. described the frailty phenotype as a clinical syndrome characterized by reduced physiological reserve and increased vulnerability to stressors, strongly associated with disability and adverse outcomes [24]. Furthermore, multimorbidity tends to increase sharply with age and contributes cumulatively to functional decline [25].

The presence of co-morbid conditions emerged as a strong independent predictor of functional dependency in this study. This aligns with substantial evidence linking multimorbidity with disability. Marengoni et al., in a systematic review, highlighted that the co-occurrence of chronic diseases significantly increases the risk of functional impairment and loss of independence [25]. Similarly, Garin et al., using multi-country SAGE data, demonstrated that multimorbidity substantially elevates the odds of ADL and IADL limitations across low- and middle-income countries [26]. Chronic non-communicable diseases such as diabetes mellitus and hypertension may lead to complications, including neuropathy, cardiovascular events, visual impairment, and reduced mobility, thereby directly affecting functional performance. These findings underscore the importance of effective chronic disease management at the primary care level to prevent or delay disability.

Educational and occupational status remained significant predictors even after adjustment in the logistic regression model. Illiterate participants had markedly higher odds of dependency compared to those with higher educational attainment. This observation is consistent with prior research demonstrating a socioeconomic gradient in disability. Zimmer and House reported that lower education is associated with a higher risk of functional limitation onset and progression, likely mediated through poorer health literacy, limited access to healthcare, and increased exposure to adverse life-course conditions [27]. Education may also influence health behaviors, preventive care utilization, and the capacity to manage chronic illness [28]. Similarly, employment or continued occupational engagement in later life may serve as a protective factor by maintaining physical activity, social interaction, and cognitive stimulation. The independent effect of female gender in the adjusted analysis further underscores the structural and socioeconomic disadvantages faced by older women, a phenomenon also highlighted in global aging reports [1,29].

The findings of this study have important public health implications. With rapid population aging in India, where the proportion of older adults is projected to rise substantially in the coming decades [2], the burden of functional dependency is expected to increase. Community-level identification of at-risk older individuals, integration of functional assessment into routine primary healthcare, and strengthening of geriatric services under the National Program for Health Care of the Elderly (NPHCE) are critical steps. Early interventions focusing on chronic disease control, physiotherapy, nutritional support, and social participation may help maintain independence and improve quality of life.

Limitations

The findings should be interpreted in light of certain limitations. The cross-sectional design precludes causal inference. The use of both probability sampling and non-probability consecutive sampling may limit generalizability. Since face-to-face interviews were used to collect data, there might have been an element of interviewer bias. Exclusion of bedridden and severely cognitively impaired individuals may have led to underestimation of true dependency prevalence. Also, we request a cautious interpretation of the association between age and functional dependency, as we experienced data separation during the analysis. Additionally, traditional gender-based scoring of the Lawton-Brody IADL scale may influence observed sex differences and may have led to underestimation in IADL dependency in males. Despite these limitations, the study contributes valuable region-specific data from an underrepresented geographical area and highlights key sociodemographic and health-related correlates of functional dependency among older adults.

## Conclusions

The results of this study show that functional dependence is a prevalent problem in Indian society. More than one-third of the study population experienced a moderate to total functional dependency. In this cohort, individuals with functional dependence shared certain sociodemographic characteristics, such as a lower level of education, unemployment, and a higher burden of morbidity, but not a lower socioeconomic status, a situation that could be associated with the socioeconomic model of this population. It was also observed that women were more likely to exhibit functional dependence, and that this dependence exhibited characteristics suggesting the persistence of gendered roles in which women were responsible for specific caregiving and household tasks, while men were responsible for economic and social activities, a finding likely associated with a traditional societal model. The prevalence of functional dependence observed in those aged 70 years and above is very high, a situation that, although it must be verified in other studies, does allow us to raise the need to establish measures through health and social planning to promote healthy aging. Owing to the rapid increase in our aging population, there is a need to emphasize the primary preventive measures and early diagnosis and treatment of chronic diseases, particularly those associated with physical disability. This also underscores the need for good-quality home care and geriatric health care services at the primary, secondary and tertiary levels. Functional assessment of older persons should be incorporated into routine patient screening. It would help evaluate the individual's health status and indicate the interventions needed if it declines.

## Appendices

**Table 9:** Participant distribution of diagnosed co-morbid conditions *Multiple responses allowed per participant

Co-morbidity^*^	Total	Males (%)	Females (%)
Arthritis	7	0	7 (100%)
Diabetes mellitus	37	7 (18.91%)	30 (81.08%)
Hypertension	156	65 (41.66%)	91 (58.33%)
Hypothyroidism	95	38 (40.00%)	57 (60.00%)
Osteoporosis	54	19 (35.18%)	35 (64.81%)
